# A Molecular Analysis of Mutations at the Complex *dumpy* Locus in *Drosophila melanogaster*

**DOI:** 10.1371/journal.pone.0012319

**Published:** 2010-08-23

**Authors:** Amber Carmon, Michael J. Guertin, Olga Grushko, Brad Marshall, Ross MacIntyre

**Affiliations:** 1 Department of Molecular Biology and Genetics, Cornell University, Ithaca, New York, United States of America; 2 Life Sciences Institute, University of Michigan, Ann Arbor, Michigan, United States of America; VIB, Belgium

## Abstract

The Drosophila *dumpy* gene consists of seventy eight coding exons and encodes a huge extracellular matrix protein containing large numbers of epidermal growth factor-like (EGF) modules and a novel module called dumpy (DPY). A molecular analysis of forty five mutations in the *dumpy* gene of *Drosophila melanogaster* was carried out. Mutations in this gene affect three phenotypes: wing shape, thoracic cuticular defects, and lethality. Most of the mutations were chemically induced in a single *dumpy* allele and were analyzed using a nuclease that cleaves single base pair mismatches in reannealed duplexes followed by dHPLC. Additionally, several spontaneous mutations were analyzed. Virtually all of the chemically induced mutations, except for several in a single exon, either generate nonsense codons or lesions that result in downstream stop codons in the reading frame. The remaining chemically induced mutations remove splice sites in the nascent *dumpy* message. We propose that the vast majority of nonsense mutations that affect all three basic *dumpy* phenotypes are in constitutive exons, whereas nonsense mutants that remove only one or two of the basic functions are in alternatively spliced exons. Evolutionary comparisons of the *dumpy* gene from seven Drosophila species show strong conservation of the 5′ ends of exons where mutants with partial *dumpy* function are found. In addition, reverse transcription PCR analyses reveal transcripts in which exons marked by nonsense mutations with partial *dumpy* function are absent.

*“All things considered, it is better to have a mutant than not to have a mutant.”*

Gerry Finkca 1970

## Introduction

The history of the *dumpy* gene in *Drosophila melanogaster* encompasses virtually the entire history of Drosophila genetics itself. Early last century, several mutants which initially seemed to have different phenotypes were recovered by the Morgan lab at Columbia University. Morgan himself noticed a fly in August of 1910 with shortened wings which he called Truncate [1]–[3]. A fly with pits on the thorax and whorls of the bristles was found in 1916 and termed vortex-II due to its location on the second chromosome [4]. In 1918 [5] a fly was discovered with both shortened wings and with whorls of bristles and hairs on the thorax. This mutant was called dumpy, the first time this term was used. These mutants, along with a second vortex mutant and another mutant named thoraxate showing thoracic vortices and homozygous lethality, were eventually combined by Bridges and Muller as a series of recessive allelomorphs possibly occurring in different parts of a single gene [6]–[8]. In the 1950s, Elof Carlson, then at UCLA, and his students generated a large number of *dumpy* mutant alleles, primarily with chemical mutagens [9]–[13]. A genetic fine structure map with discrete subloci was developed culminating in the map published by Dale Grace in 1980 [14]–[17].

Beginning in the middle of the last century, Drosophila geneticists defined and analyzed a number of complex loci. Like *dumpy*, these genes were characterized primarily by mutations with different and sometimes overlapping phenotypes, complex patterns of complementation, and genetic fine structure maps exhibiting separable clusters of mutant sites called subloci. With the advent of molecular cloning and sequencing, the underlying basis for the phenotypic complexity and the complementation patterns of many, if not most, of those loci could be explained. In addition, different functions could be assigned to groups of mutant alleles mapping at discrete subloci in fine structure maps of the genes. Two genes where cloning and sequencing provided explanations for their complexity are *rudimentary*, where complementing mutants affect distinct domains in the protein [18] and *cut*, where complementing mutants map either in the regulatory region or in the coding exons of the gene [19]. In contrast, the complexity of the *dumpy* gene in *Drosophila melanogaster*, despite being cloned and sequenced [20], has remained unexplained.

**Figure 1:**
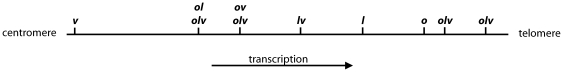
Fine structure genetic map of the subloci in the *dumpy* gene based on the mutants examined in this study (adapted from Grace [**17**]). The map is drawn approximately to scale in terms of recombinational distances. Classes of *dumpy* mutant alleles found at each sublocus are shown above the line, and the direction of transcription is shown below the map. *Dp* ^olv^ mutations are found at many sites throughout the gene.

Recessive mutant alleles of *dumpy* have three primary effects: oblique (*dp* ^o^) that affect the shape of the wing, vortex (*dp* ^v^) that disrupt the attachments of indirect flight muscle to the dorsal thoracic cuticle causing pits and protrusions, and *lethal* (*dp* ^l^) acting mostly at larval moults. The oblique and vortex phenotypes are shown in Figure 1, b and d respectively from Wilkin et al. [20].

Pleiotropic individual alleles of *dumpy*, shown in Table 1, can exhibit any combination of the three mutant phenes, and heteroallelic heterozygotes will show the phenotype of the homozygous “alleles”, e.g. *dp* ^olv^ /*dp* ^v^ flies will be viable with normal wings but mutant for vortex.

**Table 1:** Phenotypes and complementation patterns of the classes of *dumpy* mutant alleles.

Allele	Phenotype	Phenotypes of heterozygotes						
		*dp* ^o^	*dp* ^v^	*dp* ^l^	*dp* ^ov^	*dp* ^ol^	*dp* ^lv^	*dp* ^olv^
*dp* ^o^	oblique	O	+	+	O	O	+	O
*dp* ^v^	vortex		V	+	V	+	V	V
*dp* ^l^	lethal			L	+	L	L	L*
*dp* ^ov^	oblique, vortex				OV	O	V	OV
*dp* ^ol^	oblique, lethal					L	L*	L*
*dp* ^lv^	vortex, lethal						L	L*
*dp* ^olv^	oblique, lethal, vortex							L*

+ = wild type, O = oblique wings, V = vortex, L = lethal.

*may show interallelic complementation for lethality.

Importantly, there are also cases of intragenic or interallelic complementation between some *dp* ^ol^, *dp* ^lv^, *dp* ^l^, and *dp* ^olv^ alleles—marked with an asterisk in Table 1—revealing additional genetic complexity, presumably reflecting different biological roles for *dumpy* at different developmental stages.

The large size of the *dumpy* gene (the largest euchromatic gene in Drosophila) has made the construction of fine structure maps of the locus feasible. A detailed map—adapted from Grace's paper [17] to include just the mutants analyzed in this study —is shown in Figure 1. Note that *dp* ^ol^, *dp* ^ov^, *dp* ^v^, *dp* ^lv^, and *dp* ^l^, alleles occupy recombinationally distinct subloci, whereas *dp* ^olv^ alleles are found throughout the locus. In Grace's original genetic map, *dp* ^o^ alleles also mapped at several places in the gene.

**Figure 2:**
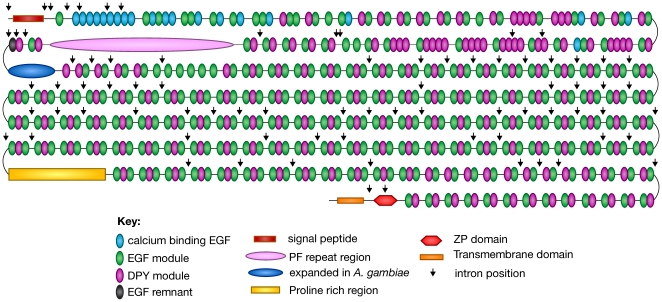
Modular structure of the *dumpy* gene product. Adapted from Wilkin et al. [20]. Modules are designated as shown in the key. Note that a large part of the protein is composed of EGF-DPY-EGF triads, with two repeated regions containing PIGSFEAST (PF) and Proline rich repeats respectively. The N-terminus is enriched in calcium binding EGF repeats and the C-terminus contains a transmembrane domain and a Zona Pellucida (ZP) domain. The arrowheads denote the positions of introns in the gene.

As shown in Figure 2, *dumpy* encodes a large protein comprised of more than 300 epidermal growth factor (EGF) repeats, a class of modules found in many extracellular matrix (ECM) proteins. Most of the EGF modules are interspersed with a novel repeat of 21 amino acids, which we have termed the DPY module, and much of the Dumpy protein is composed of contiguous repeats of a three-module EGF-DPY-EGF unit. The EGF-DPY-EGF repeats are interrupted by an insert of a repetitive, proline-rich sequence (PR) and by approximately 40 tandem, nearly identical copies of a novel 102 amino acid repeat which we call “PIGSFEAST” (PF) (since the single letter amino acid code of the repeat contains these two “words”). Our lab recently showed the PIGSFEAST region is evolving in a concerted fashion, most likely by unequal crossing over [21], [22]. Near Dumpy's N-terminus are found copies of a sub-class of Ca^2+^ binding EGF modules, and near its C-terminus there is a single Zona Pellucida (ZP) domain, found in a number of important ECM proteins where they mediate homotypic and heterotypic covalent crosslinking to other ZP domains.

Dumpy, along with two other ZP domain proteins, Piopio and Papilotte, function in the adhesion of the apical surface of the Drosophila wing epithelia to the overlying cuticle, and loss of function of each of these three genes results in a blistering phenotype in the wing [23], [24]. A further role of Dumpy in cuticle adhesion is revealed by certain larval lethal *dumpy* mutations that fail to molt due to a failure of cuticle detachment rather than a failure of adhesion. Dumpy also plays an important role in the epithelial cells that mediate the attachment of the muscles to the overlying cuticle. As mentioned above, *dumpy* vortex (*dp* ^v^) mutations result in depressions or pits in the cuticle where it overlies the muscle attachment sites. During embryogenesis, *dumpy* is expressed in many tube-forming structures that form an apical ECM that lines their internal lumens. These include the salivary gland, fore and hind-gut, and developing trachea [20]. Certain embryonic lethal *dumpy* mutations result in failure of tracheal cells in the small vessels to connect to form tubes [25]. The effect of *dumpy* mutations on the trachea may be responsible for the lethal phenotype of *dp* ^l^, *dp* ^ol^, *dp* ^lv^, and *dp* ^olv^ mutations. Hence, Dumpy has functionally diverse roles including cell adhesion, ECM assembly and mechanical properties, morphogenesis and tube formation, and as a ZP domain containing protein may interact with and modulate developmental signaling pathways [25], [26].

In this paper, we identify the molecular lesions responsible for some 45 *dumpy* mutants including examples of each kind of mutant allele shown in Table 1, and those that either complement or fail to complement other alleles. We report in this paper that most *dumpy* mutants are directly due to or lead to downstream nonsense codons, even when the mutation disrupts only one or two of the three basic mutant phenotypes. We propose that such mutants mark alternatively spliced exons whereas mutants which affect all three phenotypes (*dp* ^olv^) are located in constitutive exons. We provide some experimental evidence for this hypothesis using RT-PCR analyses. We also discuss the possibility that the complementation of certain *dp* ^olv^ mutations results from *trans*-splicing. Hence, alternative *cis* and *trans*-splicing events generating different and perhaps tissue specific Dumpy isoforms can provide a rationale for the complexity of this long studied Drosophila gene.

## Results

### Properties of chemically induced *dumpy* mutations

The crosses employed in the screens for EMS induced *dumpy* mutations in defined isoallelic backgrounds are outlined in Table 2. The distribution of mutations from crosses 1, 2A, and 2B is as follows: 60 *dp* ^olv^, 32 *dp* ^ol^, 7 *dp* ^lv^, 2 *dp* ^ov^, and 2 are *dp* ^o^. Like Jenkins [13], the majority of our mutants were *dp* ^olv^. However, in our case *dp* ^ol^ mutants outnumbered *dp* ^lv^ mutants. From the screens depicted in crosses 2A and 2B in Table 2, we recovered 90 transmitted *dumpy* mutants, 46 from cross 2A in the *net* chromosome and 44 from cross 2B in the *clot* chromosome. All of these mutations are in an identical *dumpy* allele derived from an isofemale line from Australia. The mutants, along with the flanking visible marker, their *dumpy* phenotypes, and the balancer chromosomes are listed in Table S1. The cross schemes followed in Table 2 also allowed us to detect *dumpy* lethal alleles which complement the *dp* ^lvI^ mutation in the CyO balancer chromosome.

**Table 2:** Crosses used to produce *dumpy* mutants in defined chromosomal backgrounds.

Cross	Mutagenized males	Females	F_1_ phenotype
1	*cn bw*- 2^nd^ chromosome isogenic	*dp* ^ov^ *cl*	oblique, vortex
2A	*net*, *dp* ^+^ isoallele*	*net dp* ^ov^ *cl*	oblique, vortex
2B	*cl*, *dp* ^+^ isoallele	*dp* ^ov^ *cn bw*	oblique, vortex
3	*cl*; *e*(*dp* ^v^) *dp* ^+^ isoallele	*dp* ^v1^; *e*(*dp* ^v^)	vortex
4	*net*; *dp* ^+^ isoallele	In(2LR) *Gla*/*dp* ^lv^ *cl*; *e*(*dp* ^v^)	vortex

*A single *dp* ^+^ allele isolated from a wild type strain collected in Australia. See text for details.

We initiated the screens designated as crosses 3 and 4 in Table 2 to enrich for *dumpy* vortex mutants, since none was recovered from crosses 1 and 2. Cross 3, in which F1 males carrying the *dp* ^v1^ mutant from the Bloomington stock collection in the presence of the *e*(*dp* ^v^) mutation on the 3^rd^ chromosome were scored, produced eleven *dp* ^olv^ mutations, one which complements *dp* ^lvI^, and two new *dp* ^lv^ alleles. These are listed in Table S1 as *dp* ^olvRX^ or *dp* ^lvRX^ respectively. Since alleles with oblique phenotypes also came through this screen, we set up cross 4 in Table 2, this time examining F1 males carrying a previously generated *dp* ^lv^ allele and homozygous for *e*(*dp* ^v^). 24,000 F1s were scored and four complementing *dp* ^olv^ mutants were obtained, along with a single *dp* ^ov^ mutant allele. Hence this screen appears to enrich for complementing *dp* ^olv^ mutations. We will discuss below how such mutations may help identify putative *trans*-splicing events in the *dumpy* gene.

### Molecular basis of *dumpy* mutations

**Figure 3:**
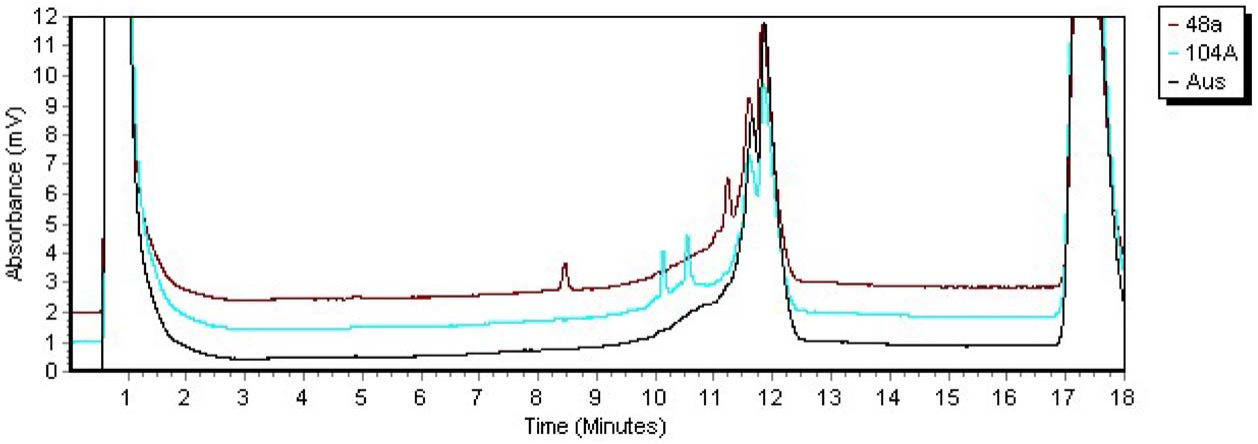
dHPLC patterns of two *dumpy* mutants, *dp* ^48a^ and *dp* ^104A^, located in exon 11 and the wild type progenitor allele from Australia. The elution of the two cleavage fragments generated by the Surveyor nuclease from each mutant are shown in dark red for *dp* ^48a^ and light blue for *dp* ^104A^.

We outline in the Materials and Methods section the approaches we've taken to characterize preexisting *dumpy* mutations, those we generated in strains isoallelic for a wild type *dumpy* allele from Australia, and those recovered in a screen for spontaneous mutations. Most of these analyses relied on the generation of overlapping amplicons across the entire gene and the use of the WAVE dHPLC machine from Transgenomic, Inc. to detect cleavage fragments generated by the Surveyor nuclease at the sites of base pair mismatches. A typical dHPLC chromatogram is shown in Figure 3, where two mutations *dp* ^olv48a^ and *dp* ^olv104A^ are located in an amplicon from exon 11.

**Figure 4:**
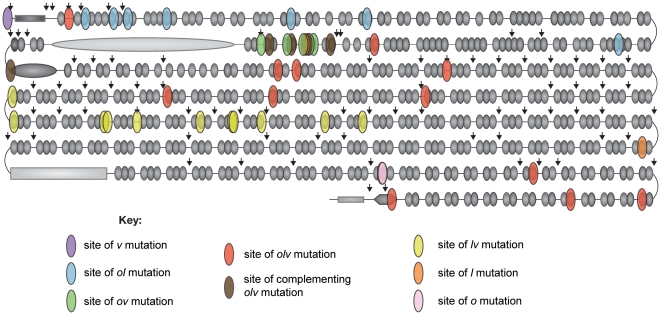
Positions of the sequenced *dumpy* mutations in the gene product as diagrammed in **Figure 2**. Note the regional localization of *dp* ^v^ (purple), *dp* ^ol^ (blue), *dp* ^ov^ (green), complementing *dp* ^olv^ (brown), *dp* ^lv^ (yellow), *dp* ^l^ (orange), and *dp* ^o^ (pink) mutations. *Dp* ^olv^ mutations (red) are located at several different sites in the protein.

Table 3 and Figure 4 show our results to date. Clearly this approach is very effective in detecting and identifying mutations in the *dumpy* gene. The data are remarkable in that most of the mutations, including *dp* ^ol^ and *dp* ^lv^ mutants, result in stop codons either at the site of the mutation, are generated from a deletion, or cause the removal of a splice site. It is interesting that all missense mutations identified to date change cysteines in the protein. Given the repetitive nature of the Dumpy protein, i.e. all the EGF-DPY-EGF motifs, perhaps most missense mutations don't produce a visible phenotype.

**Table 3:** Results of molecular analyses of selected *dumpy* mutations.

Mutant	Origin	Allele Class	Exon	Mutation or Deletion	Effect
v2	SC	v	5′ region	roo transposon	unknown
1C5	BM	olv	3	G->A	removes splice site
36a	MG	ol	5	TGC->TAC	Cys->Tyr
38a	MG	ol	6	2bp deletion	frameshift and stops
67b	MG	ol	7	TGT->TGA	Cys->STOP
2P1	BM	ol	7	CAA->TAA	Gln->STOP
71a	MG	ol	7	TGG->TAG	Trp->STOP
18b	MG	ol	7	TCG->TAG	Ser->STOP
D1311A	OG	ol	7	15bp inversion	creates a STOP
2G1A	BM	olv	9	CAA->TAA	Gln->STOP
105A	MG	olv	11	CAA->TAA	Gln->STOP
56a	MG	ov	11	TGT->TAT	Cys->Tyr
7b	MG	ov	11	TGC->CGC	Cys->Arg
104A	MG	olv	11	TGC->AGC	Cys->Ser
61B	MG	olv	11	TGT->CGT	Cys->Arg
A12	RM	ov	11	TGC->TAC	Cys->Tyr
27B	MG	olv	11	TGC->TAC	Cys->Tyr
ovDG2	SC	ov	11	TGT->TAT	Cys->Tyr
48a	MG	olv	11	TGC->TCC	Cys->Ser
ov1	SC	ov	Intron 11	blood transposon	unknown
6	MG	olv	15	4bp deletion	frameshift->STOP
R11	RM	olv	19	CGA ->TGA	Arg->STOP
D2011A	OG	olv	19	16bp deletion	frameshift->STOP
G8202B	OG	olv	Intron 21	6bp deletion	unknown
R4	RM	olv	33	AGA->TGA	Arg->STOP
2C1	BM	olv	34	TGT->TGA	Cys->STOP
89a	MG	olv	34	CAG->TAG	Gln->STOP
G3030B	OG	lv	40	89bp deletion	frameshift ->STOP
L2311B	OG	lv	40	TAC->TAA	Tyr->STOP
23b	MG	lv	43	368bp deletion	frameshift
H1230B	OG	lv	43 to 45	1140bp deletion	frameshift
lvR2	RM	lv	43	G->A	removes splice site
16	MP	lv	45	10bp deletion	frameshift and stops
12	MP	lv	46	139bp deletion	removes splice site
7a	MG	lv	46	CAG->TAG	Gln->STOP
D1191A	OG	lv	47	CAA->TAA	Gln->STOP
65f	MG	lv	48	CAG->TAG	Gln->STOP
P1129B	OG	lv	49	1bp deletion	frameshift
lDG82	SC	l	58	GAG->TAG	Glu->STOP
o2	SC	o	72	TGT->TAT	Cys->Tyr
o14b	MG	o	72	TGT->TAT	Cys->Tyr
5B1	BM	olv	73	CAA->TAA	Gln->STOP
12B1	BM	olv	76	1482bp deletion	unknown
21C2	BM	olv	76	CAG->TAG	Gln->STOP
R3	RM	olv	76	CAG->TAG	Gln->STOP

The *dp* ^olv^ mutants are found scattered throughout the locus. Again, most identified so far introduce a stop codon or otherwise lead to a truncation of the protein via a frameshift, or remove a splice junction. It should be noted that this leads to the same severe class of phenotype whether the predicted molecular product is a short N-terminal region or includes the majority of the extracellular domain. It might be expected that generating such a long fragment of the protein would allow the mutant to act as a dominant negative. However, all of the *dp* ^olv^ mutants behave as straightforward recessive, loss of function alleles. This implies that, to retain any function, the product must translate through to the C-terminus.

In contrast to the *dp* ^olv^ mutant sites which are located at many different places in the gene, an observation that is consistent with their many sites in Grace's fine structure map [17], most of the other *dumpy* mutants are found clustered in discrete regions. We will discuss each of these clusters, proceeding from the 5′ to the 3′ end of the gene.

### *dp* ^v^ mutants

To date, we've not recovered any EMS induced vortex mutants in the *dumpy* allele in 2^nd^ chromosomes marked with the *net* and *clot* mutants. We did, however, examine pre-existing *dp* ^v^ alleles, *dp* ^v1^ and *dp* ^v2^ [4], [7], [8]. Both were originally recovered in the Morgan laboratory at Columbia University early last century. Using the primers listed in Table S2, we generated amplicons across the entire gene from each homozygous vortex mutant except with primers 5′19F and 5′19R. We then used long range PCR (see Materials and Methods) and recovered amplicons approximately 10kb in length from each mutant. Sequences from the ends of these amplicons indicated the presence of a *roo* element at bp 15448 upstream of the start codon, in the 5′ region of the gene in both alleles. If the *roo* element is responsible for the *vortex* mutant phenotype, its position in the gene is consistent with the position of the vortex sublocus in Grace's fine structure map. We will discuss the identity of the two supposedly independently isolated alleles below.

### *dp* ^ol^ mutants

Note in Table 3 that these seven mutations localize to exons 5, 6, and 7 near the N-terminus of the protein, again consistent with the position of the *ol* sublocus in Grace's map. Except for *dp* ^ol36a^, these mutations produce or result in a stop codon. *Dp* ^ol36a^ is a missense mutation in which a cysteine residue in a Ca^2+^ binding EGF motif is replaced with a tyrosine. The cysteine residues in these short motifs (ca 35 amino acids) are essential for their correct tertiary structure due to their participation in disulfide bonds [20]. The position of this missense mutation is in exon 5 and is the *dp* ^ol^ mutant closest to the 5′ end of the gene (see Figure 4).

### *dp* ^ov^ mutants

All of these mutations are in exon 11 or in the adjacent intron. The mutant *dp* ^ov1^, discovered by Morgan in 1918 [5], has been the canonical *dumpy* mutant used by many investigators in the last century. It is characterized by full penetrance and intermediate expressivity, e.g. virtually all females in *dp* ^ov1^ containing stocks have oblique wings with an intermediate score of 3 on Dale Grace's scale [17], [27]. Males exhibit a lower penetrance and lower oblique scores on the Grace scale. We examined *dp* ^ov1^ after finding the EMS induced *dp* ^ov^ mutations in the amplicon from exon 11 by sequencing. No nonsynonymous changes were found nor any changes affecting the canonical splice sites. Intron 10 and intron 11 were then sequenced. The primers used to amplify a region of intron 11 failed to produce a product from *dp* ^ov1^ DNA. Long range PCR, however, did produce a product containing a *blood* transposon. This insertion is in the intron just preceding the large exon encoding the PIGSFEAST repeats that are undergoing concerted evolution [21], [22]. The EMS induced *dp* ^ov^ mutants, including *dp* ^ovDG2^ from Dale Grace, are missense mutants affecting cysteine residues in the EGF-DPY diads that characterize exon 11. Curiously, except for *dp* ^ol36a^ which affects exon 5 and both *dp* ^o^ mutants affecting exon 72, exon 11 is the only other exon in which missense mutations have been recovered.

### *dp* ^lv^ mutants

We have characterized 11 *dp* ^lv^ mutations and most either directly generate a stop codon or are out of frame deletions (see Table 3). The EMS generated mutant, *dp* ^lvR2^, is a G to A transition removing a splice site between exons 43 and 44, and none of the *dp* ^lv^ mutants is due to an amino acid substitution, although the region of the protein affected, *viz*. exons 40–49 consists of consecutive repeats of EGF-DPY-EGF triad domains whose tertiary structures are surely stabilized by cysteines participating in disulfide bonds (see Figure 3c in Wilkin et al. [20]). Note also that there are no *dp* ^olv^ mutations located in the *dp* ^lv^ region, nor did Grace map any *dp* ^olv^ mutations in the *lv* sublocus. Once again the positions of the *dp* ^lv^ mutations in the protein are colinear with the position of the *lv* sublocus in Grace's map.

### *dp* ^l^ mutant

We did not recover any *dp* ^l^ mutants in our screen for EMS induced mutants, nor did we expect to given the design of the cross scheme. We screened F1s for oblique and/or vortex phenotypes over *dp* ^ov1^ or *dp* ^lvR1^ alleles. Indeed, it is difficult to envision an F1 screen for mosaics which would allow for the recovery of *dp* ^l^ mutations. A search of the literature including Masters and PhD theses did not reveal how such mutants were recovered. Nevertheless, we were able to obtain two mutants, *dp* ^lDG82^ and *dp* ^lDG83^, from the Kyoto stock center induced by Dale Grace [15], [17]. Crosses with these mutually non-complementing mutants do indeed confirm their status as *dp* ^l^ mutants, i.e. they produce wild type F1 adults when crossed to *dp* ^o^, *dp* ^v^, or *dp* ^ov^ mutants and F1s from crosses to *dp* ^ol^, *dp* ^lv^, and most *dp* ^olv^ flies do not survive to adulthood (see Table 1). We determined that *dp* ^lDG82^ is due to a nonsense mutation in exon 58. The *dp* ^l^ mutant, identified as distinct recombinationally from the *lv* sublocus, is also molecularly discrete from the exons marked by *dp* ^lv^ mutations.

### *dp* ^olv^ mutants

Grace mapped *dp* ^olv^ mutations at many different sites in the gene, and we also find these mutants at many different places in the Dumpy protein. For example, *dp* ^olv1C5^ affects the 3^rd^ exon, *dp* ^olv2C1^ and *dp* ^olv89a^ are both stop codons in exon 34, whereas *dp* ^olv21C2^ and *dp* ^olvR3^ are due to nonsense codons in exon 76 which encodes the ZP domain very near the C-terminus. Except for those mutations in exon 11 and *dp* ^olv1C5^, which results in the removal of a splice site, *dp* ^olv^ mutants result from either stop codons or deletions, which generate frameshifts and downstream stop codons. *Dp* ^olv12B1^ is a very large in frame deletion which removes a large portion of exon 76. Again, in agreement with Grace's genetic map, no *dp* ^olv^ mutations are found in the exons of the *ol*, *lv*, or *l* subloci. The *ov* sublocus, presumably encompassing only exon 11 and an adjacent intron, is another story. Here Grace mapped *dp* ^ov^, *dp* ^olv^, and *dp* ^o^ mutations at the same site, given the limited resolving power of recombination in a higher eukaryote such as Drosophila. We too find both *dp* ^olv^ and *dp* ^ov^ mutations in exon 11, but curiously, and except for *dp* ^olv105A^, the *dp* ^olv^ mutations in this exon and only this exon are missense mutations, all four of which substitute a different amino acid for a cysteine residue. We will discuss below how *dumpy* mutations with several different phenotypes could be found in a single exon. We have also observed that certain *dp* ^olv^ mutations will complement other *dumpy* lethal alleles, particularly other *dp* ^lv^ and *dp* ^ol^ mutations. In these cases, the surviving F1s show good viability but will exhibit vortices or have oblique wings respectively. In these cases the complementing mutations result from a stop codon. We also find cases of complementation between different *dp* ^olv^ mutants for example, *dp* ^olv104A^/*dp* ^olv6^ F1s are fully viable but have vortices and oblique wings. Note in Figure 4 that complementing *dp* ^olv^ mutants we have analyzed appear to closely flank the highly repeated PIGSFEAST region, and indeed, all but *dp* ^olv6^ are located in exon 11. Three of the complementing *dp* ^olv^ mutants in exon 11 are missense mutations but the complementing mutant *dp* ^olv105A^ is due to a nonsense mutation, and *dp* ^olv6^ in exon 15 on the other side of the PIGSFEAST exon is a frameshift mutation which generates a stop codon.

### *dp* ^o^ mutants

Our sample of sequenced mutants is deficient for oblique or *dp* ^o^ alleles. These unfortunately are only rarely recovered in EMS screens , although Grace found that, like *dp* ^olv^ mutants, they map at many places in the gene. We did analyze two *dp* ^o^ mutations. Both are missense mutations that, remarkably, are due to G to A transitions of the same nucleotide resulting in cysteine to tyrosine substitutions. We are certain these are different mutations since the SNP patterns and synonymous substitutions in the chromosomes surrounding the site are very different.

### Alternative splicing in Dumpy: Evolutionary evidence

Our molecular analyses of the *dumpy* mutants indicates most are due to nonsense mutations. One might predict, if *dumpy* encodes a single transcript and translated message, that most, if not all, of these would affect all three basic functions, i.e. wing shape, tendon cell-cuticle attachment and ultimately viability. Hence they should have a *dp* ^olv^ phenotype. How then do we explain the observations that the *dp* ^ol^, *dp* ^lv^, and *dp* ^l^mutants, i.e. those that have only partial *dumpy* function, are also due to the presence of stop codons in the *dumpy* message? We propose that these mutations producing partial functions will be found in alternatively spliced exons. For example, exons tagged by *dp* ^lv^ nonsense mutations will be expressed in certain tissues, e.g. in tendon cells and in the trachea, the latter presumably necessary for viability, but not be present in *dumpy* messages in the developing wing. *Dp* ^olv^ nonsense mutants would be found in so called constitutive exons expressed in most, if not all, tissues at all developmental stages. Other explanations of our results are discussed below.

**Figure 5:**
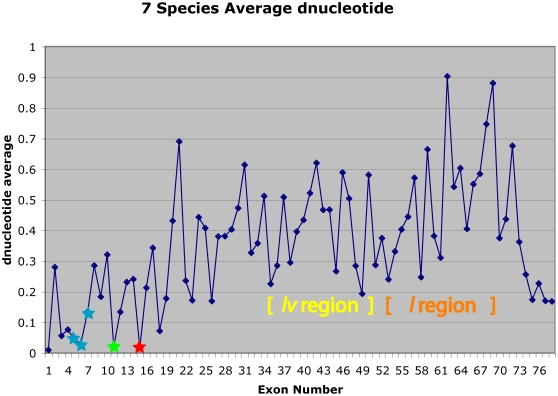
Graph of average d_nucleotide_ differences between the first 30 sites at the 5′ ends of *dumpy* exons from seven Drosophila species. There is strong conservation of the sequences at the 5′ ends of *ol* exons (blue stars), exon 11 (green star), and exon 15 containing the complementing mutation *dp* ^olv6^ (red star). In general, exons containing other *dp* ^olv^ mutations show higher levels of divergence. See Materials and Methods for details regarding alignment and computational procedures.

The hypothesis that the *dumpy* gene encodes both alternative and constitutive exons makes several predications. First, there are distinct differences between alternative and constitutive exons in other systems. Xing and Lee [28], [29] noted that RNA sequences from alternatively spliced exon/intron boundaries leads to selection pressure for nucleotide sequence conservation in these regions while there is significantly less conservation in constitutive exons. Thus, they noted that K_s_, the number of synonymous substitutions per synonymous site, is much lower in human-mouse comparisons of alternatively spliced exons than in constitutive exons. To assess sequence divergence in *dumpy*'s exons, we compared the first 30 nucleotides of the exons from seven Drosophila species (*D. melanogaster*, *D. ananassae*, *D. pseudoobscura*, *D. willistoni*, *D. mojavensis*, *D. virilis*, and *D. grimshawi*), as shown in Figure 5.

It should be noted that the seven species compared in a pairwise fashion (the averages are shown in Figure 5) are, in each case, from different subgenera or from different species groups. The conservation of the 5′ ends of exons marked by *dp* ^ol^ (blue stars) mutations is striking. Exon 11 (green star) and exon 15 (red star), both of which are affected by complementing *dp* ^olv^ mutations, are also striking in their conservation of 5′ nucleotides. The nearly complete sequence identity of the 5′ ends of these exons over 60 million years of evolutionary time indicates there is a highly conserved interaction between the *dumpy* message from these regions and proteins involved in the splicing process, possibly in a tissue specific manner [30]. The pattern of conservation in the *lv* and *l* regions is also very interesting (bracketed yellow and orange areas). There may be several different mechanisms creating various alternative transcripts in the *lv* and *l* region such as competing intronic RNA secondary structures [31], steric hinderance of multiple splicing factor binding sites, or major and minor splicesome usage [32]. In general, exons with non complementing *dp* ^olv^ mutations do not show marked 5′ end conservation. For example, exons 19 and 34 show higher levels of nucleotide divergence *viz*. 0.178 and 0.512 respectively and each is marked by two non complementing *dp* ^olv^ nonsense mutations, *dp* ^olvR11^ and *dp* ^olvD2011A^ in exon 19, and *dp* ^olv2C1^ and *dp* ^olv89a^ in exon 34. As in the case of the human-mouse comparisons [28], [29], there is marked conservation in some alternatively spliced exons.

### Alternative splicing in Dumpy: Evidence from RT-PCR

We have evidence that alternatively spliced *dumpy* mRNAs can be detected by RT-PCR. We extracted mRNA from wild type 3^rd^ instar larvae and from *Drosophila* S2 cell lines and used primers spanning the set of *ol* and *lv* exons. Primers spanning the two exons of the ZP domain were used as a positive control. We also chose primers located in exons marked by *dp* ^olv^ nonsense mutations that we believe are constitutive.

**Figure 6:**
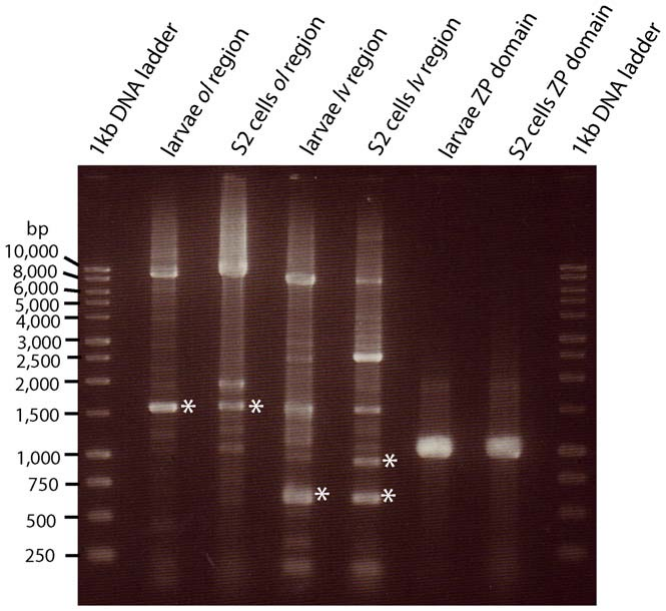
Gel showing RT-PCR products from the *dumpy* gene in 3^rd^ instar larvae and S2 cells. Primers flanking the *ol* and *lv* regions and the ZP domain that were used to generate these products are shown in Table S2. Bands marked with asterisks were excised and sequenced.

**Figure 7:**
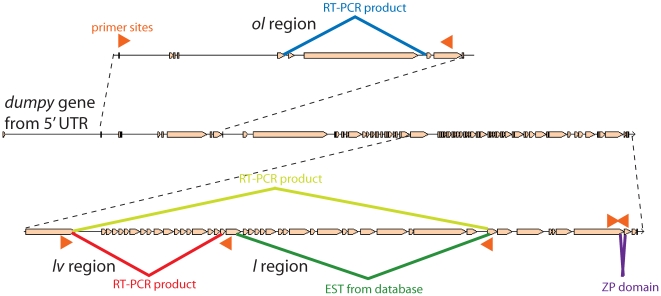
Diagram of RT-PCR products exhibiting alternative splicing in the *dumpy* gene. The middle line shows the intron-exon structure of the wild type *dumpy* gene. The *ol* region is shown above the line and the RT-PCR product which is missing exons 6 and 7. Below the line depicting the wild type gene is the intron-exon structure of the 3′ end of the gene showing the *lv* and *l* regions and the ZP domain. The RT-PCR products are missing a number of exons from each region. In each case, arrowheads mark the positions of primers used to obtain the RT-PCR products. These primer sequences are shown in Table S2.

Figure 6 is a gel image of the RT-PCR products obtained from the two sources of mRNA. There are a number of shorter products, some of which were sequenced (identified by asterisks in Figure 6). Sequence data clearly indicate the presence of alternatively spliced mRNAs in both 3^rd^ instar larvae and S2 cells. The primers spanning the *ol* region detected a mRNA lacking exons 6 and 7 diagrammed in Figure 7. Recall that 6 of the 7 *dp* ^ol^ nonsense mutations are in these two exons. At least two differently spliced messages were obtained using the primers spanning the *lv* exons, one is missing exons 35 to 50 where all of the *dp* ^lv^ mutations are located, whereas the other skips a larger number of exons, 35 to 69. The largest band in each is consistent with inclusion of all exons between the selected primers and illustrates we can amplify at least 10kb by RT-PCR. The intermediately sized bands most likely represent different alternatively spliced products with various exons included in the transcripts.

The Flybase Drosophila EST database (http://flybase.org) for *dumpy* is highly enriched for clones with sequences from the 3′ end of the gene and essentially is non informative with regard to alternative splicing patterns. There is one EST from the database, however, that excludes exons 53 to 69 (see Figure 7). Recall that the *dp* ^lDG82^ nonsense mutation is in exon 58, and according to our hypothesis this exon should be alternatively spliced.

## Discussion

Our attempts to generate new *dumpy* mutants in an isoallelic 2^nd^ chromosome with ethyl methanesulfonate produced a distribution of *dumpy* alleles similar to that of Jenkins [13] Table 5. Thus the majority of our mutants generated in crosses 1 and 2 (see Table S1) were *dp* ^olv^, *dp* ^ol^, and *dp* ^lv^ in decreasing order. Other chemical mutagens – see Table 6 in Jenkins [13]- produce similar distributions. All of these cross schemes involved screening F1s heterozygous for *dp* ^ov1^ for oblique wing phenotypes and/or thoracic vortices. It's possible that the greater ease with which oblique versus vortex phenotypes are detected could bias such screens utilizing *dp* ^ov1^ toward the recovery of *dp* ^olv^, *dp* ^ol^, *dp* ^ov^, and *dp* ^o^ mutants, yet the latter two types are recovered very infrequently. Hence we feel the distribution of mutants accurately reflects the sizes of “targets” within the *dumpy* gene which, when mutant, affect one or more combinations of phenotypes. We are collaborating with the laboratory of Olga Grushko and Alexey Kondrashov at the University of Michigan where spontaneous *dumpy* mutants are being isolated as non-fliers at 28°C. To date, eight such mutations have been analyzed molecularly (see Table 3). As with the EMS induced mutants, lethal classes predominate and in this case they are either *dp* ^ol^ (1), *dp* ^olv^ (2) or *dp* ^lv^ (5) mutants. Except for two mutants, *dp* ^L2311B^ and *dp* ^lvD1191A^, all of the others are deletions or small inversions which create frame shifts and/or stop codons. There is also a deletion in an intron with an unknown effect.

Due to our failure to recover *dp* ^v^ mutants in the screens utilizing crosses 1 and 2 in Table 2 and because only two such alleles currently exist in stock centers, *dp* ^v1^ and *dp* ^v2^, we set up crosses 3 and 4 to enrich for new vortex mutants in the isoallelic 2^nd^ chromosome from the Australia line. Following Jenkins [13], we estimated that we would obtain one transmitted vortex mutant in 17,500 F1 flies screened (frequency of F1 *dumpy* mutants, ca 0.8% in his Table 4, times1/20 vortex mosaics or completes in his Table 5 times 15% of transmitted vortex mutations in his Table 6).

The number of F1s screened in cross 3 was not estimated, but in cross 4, in two separate mutageneses, we examined an estimated 24,000 flies. Five F1 flies with mosaic or complete vortices transmitted the mutation but none was a *dp* ^v^ mutant. Of the five mutants, four were complementing *dp* ^olv^ alleles and one was a homozygous viable *dp* ^ov^ allele. As mentioned above, there are no stocks of the *dp* ^v^ mutants obtained by Jenkins [13], or Grace [17]. Our failure to obtain such mutants with EMS makes it more likely, in our view, that the *roo* element in the 5′ end of the *dumpy* gene in mutants *dp* ^v1^ and *dp* ^v2^ is responsible for the mutant phenotype. It may also be that these alleles are, in fact, the same—the *roo* element is inserted between the same two base pairs in each case and were perhaps inadvertently isolated at different times in the Morgan laboratory and named as separate alleles. Clearly the vortex sublocus, so elegantly mapped by Grace [17] at the 5′ end of the *dumpy* gene itself, needs to be better defined mutationally. The *dumpy* lethal sublocus, currently defined by only two alleles *dp* ^lDG82^ and *dp* ^lDG83^, also needs to be further analyzed. It is not clear, however, how additional *dp* ^l^ alleles can be obtained, since F1 screens cannot be used. F2 screens would be tedious, although it might be possible to screen the progeny of F2 individuals crossed with *dp* ^olv^ flies for homozygous lethality.

The approach we have taken to define the mutations generated with EMS in isoallelic *dumpy* wild type alleles, i.e. producing overlapping amplicons from the entire locus and screening for base pair mismatches in reannealed duplexes with Surveyor nuclease and dHPLC as shown in Figure 3, has been very effective. The data are remarkable in that most of the mutations, including *dp* ^ol^ and *dp* ^lv^ mutants, result in stop codons either at the site of the mutations or are generated from a deletion, or cause the removal of a splice site. It is also clear that all eleven missense mutations change cysteines in EGF or DPY motifs, presumably altering or destabilizing their tertiary structures. Given the repetitive nature of the Dumpy protein, i.e. all the EGF-DPY-EGF motifs, perhaps most other kinds of missense mutations don't produce a visible phenotype. As shown in Table 3, eight of the eleven missense mutations are found in exon 11 which defines the *ov* sublocus in Grace's map. This is in stark contrast to the mutational spectrum in probably all other *dumpy* exons where the mutations are almost exclusively deletions or nonsense mutations. Grace mapped three kinds of oblique mutations *viz*. *dp* ^o^, *dp* ^ov^, and *dp* ^olv^ at the *ov* sublocus, and we also find both *dp^ov^* and *dp* ^olv^ mutations in exon 11. The exon is unremarkable in that it encodes 4 simple EGF-DPY diads, which, although most of the protein consists of EGF-DPY-EGF triads (see Figure 2), are also found at other positions in the protein. Six of the missense mutants are in EGF modules and two are in the DPY members of the diads. Interestingly, in the EGF module in the second diad, there are four mutations affecting four of the six cysteines. Two of the mutations exhibit a *dp* ^ov^ phenotype and two are *dp* ^olv^ mutants, one of which, *dp* ^104A^, is a complementing mutant (see below).

At this point we don't know if the *dumpy* exon 11 is alternative or constitutive since our RT-PCR experiments did not utilize primers flanking this exon. The extreme conservation found at its 5′ end (see Figure 5) indicates it is alternatively spliced, but the presence of a *dp* ^olv^ nonsense mutation (*dp* ^olv105A^) according to our hypothesis, would make exon 11 constitutive. We are currently analyzing the splice variants in *dumpy* RNA by RNA-seq. [33], [34], the results of which should clarify the status of exon 11.

As mentioned above, we believe the long standing and hitherto unexplained complexity of the *dumpy* gene can best be explained by extensive alternative splicing where *dp* ^olv^ nonsense mutations tag constitutive exons presumably located at several different places in the gene. Nonsense mutations with partial dumpy function, e.g. *dp* ^ol^ and *dp* ^lv^ mutations, will be found in alternatively spliced exons and should be more localized in the gene.

In this regard, typical characteristics of alternatively spliced exons are small size and divisibility by 3 so as not to affect the reading frame depending on their inclusion or exclusion. The *dumpy* gene contains 78 coding exons, many are very small, i.e. under 80bp, and the number of nucleotides in all but 1 internal exon is divisible by 3.

We predict and, indeed, have found that alternative splicing produces tissue specific isoforms of Dumpy encoded by at least several kinds of mRNAs. The *dp* ^ol^, *dp* ^lv^, and *dp* ^l^ nonsense mutations either could result in truncated isoforms only in the affected tissues or, if they are located in splicing protein binding sites in the exon, prevent the formation of the alternatively spliced transcript (see [35]). These two possibilities make different predictions about whether alternatively spliced transcripts would be found in *dp* ^ol^, *dp* ^lv^, and *dp* ^l^ mutants, *viz*. if the tissue specific mutation results in truncated isoforms, the alternatively spliced transcripts would be present in mutant flies but if the mutations interfere with the splicing process, the transcripts would be aberrant or absent. Thus, a comparison of RT-PCR products in tissues from wild type and mutant flies should distinguish between the two mechanisms.

How else can we explain the observation that most of the lethal classes of *dumpy* alleles, *viz*. *dp* ^ol^, *dp* ^lv^, *dp* ^l^, and *dp* ^olv^, are due to nonsense mutations? One explanation might be that some premature stop codons in the *dp* ^olv^ mutants result in nonsense mediated decay of the mRNA [36] and this completely removes all functions in the *olv* class. However, a paradox in the results is that many of the less severe *dp* ^ol^, *dp* ^lv^, and *dp* ^l^ mutations that retain some *dumpy* function also introduce premature stop codons that truncate the protein within its extracellular domain. Hence, these data do not appear compatible with the hypothesis that the *dumpy* locus generates only one molecular product. Indeed, the RT-PCR results reported here (see Figures 6 and 7) clearly show that certain exons are excluded from some *dumpy* mRNAs by alternative splicing. Hence, we propose that *dumpy* generates multiple products by alternative splicing which are specialized to particular biological functions.

As mentioned above the mutant screens outlined in cross 4, Table 2, failed to generate new *dp* ^v^ (vortex) mutants but did, however, produce four mutant alleles, *dp* ^olv13v^, *dp* ^olvA4^, *dp* ^olvB11^ and *dp* ^olvB16^. These came through the screen because they complemented the lethal phenotype associated with a *dp* ^lv^ allele. Earlier, crosses 1 and 2 produced additional complementing *dp* ^olv^ mutants, e.g. *dp* ^olv105A^, *dp* ^olv104A^, *dp* ^olv27B^, *dp* ^olv48a^, and *dp* ^olv6^. These were also recovered as complementers of the *dp* ^lvI^ allele carried on the In (2LR) CyO balancer. Also several inter se crosses have revealed additional cases of complementation between these individual *dp* ^olv^ mutant alleles. For example, *dp* ^olv104A^ and *dp* ^olv6^, mutants which flank the PIGSFEAST exon (number 12) fully complement for viability (i.e. 1/3^rd^ of the F1s from a cross between flies from balanced lethal parents survive to adulthood, but still show oblique wings and vortices. Such interallelic or intragenic complementation is generally explained by association and functional complementation between mutant protein subunits [37], but when one (e.g. in *dp* ^olv6^/*dp* ^olv104A^ heterozygotes or both alleles (e.g. in *dp* ^olv6^/*dp* ^lDG82^ heterozygotes) are nonsense mutations, a different explanation for the complementation must be found.

Complementing *dp* ^olv^ nonsense mutants, are also difficult to explain by *cis* alternative splicing. Alternative *trans*-splicing, however, could be operating in the processing of *dumpy* messages, perhaps only in certain tissues, and provide an explanation for the viability of some *dp* ^olv^ heteroallelic heterozygotes. Paradigms for putative *trans*-splicing events have been documented [38]–[42].

Our current RNA-seq approach to detect exon-exon junctions in the *dumpy* “transcriptome,” when coupled with the identification of the mutant vs. wild type codons or SNP associations in individual cDNAs should allow us to detect *trans*-splicing events. In this regard, we find a SNP, on the average, every 140 bases in the exons of the *dumpy* gene. If *trans*-splicing turns out to be responsible for the interallelic complementation between lethal *dumpy* nonsense mutants, we can begin to identify the mechanism and the gene products that are responsible for the splicing events. This can be accomplished with screens for suppressors and enhancers of the complementation patterns of different *dumpy* mutants.

In summary, the molecular analysis of 45 preexisting, spontaneous, and/or EMS induced *dumpy* mutations revealed most missense mutations were found in exon 11. All other mutations except two transposon insertions generated stop codons, were deletions, an inversion, or frameshift generated nonsense codons, even those which exhibited only one or two of the three *dumpy* mutant phenotypes. We present evolutionary and experimental evidence for *cis* alternative splicing of *dumpy* transcripts and argue that these observations, along with the distribution within the gene of nonsense mutations with different *dumpy* mutant phenotypes, makes it likely that alternative splicing underlies the genetic and phenotypic complexity of this long studied, paradigmatic Drosophila complex gene. In addition, complementation between certain *dumpy* nonsense mutant alleles can be explained by *trans*-splicing.

## Materials and Methods

### Drosophila strains

*Dumpy* mutant alleles with undefined genetic backgrounds were ordered from Bloomington or Kyoto stock centers. These are identified in Table 3 as SC. Mutants 12 and 16 were provided by Jim Fristrom and Mary Prout, and identified as MP in Table 3. They were generated by gamma ray mutagenesis and are *dp* ^olv^ or *dp* ^lv^ alleles which, when homozygous in somatic clones, give rise to wing blisters (see Prout et al. [23]). Mutants designated with an OG in Table 3 were recovered by Olga Grushko and Alexey Kondrashov in a screen for spontaneous *dumpy* mutants. They are present in two 2^nd^ chromosomes designated as A and B, extracted from natural populations near Ann Arbor, MI, and made homozygous for chromosome 2. Each has a different pattern of single nucleotide polymorphisms (SNPs) in the *dumpy* gene. The screen selects for non fliers and, hence, the mutant alleles have an oblique wing or vortex phenotype. Most of the *dumpy* mutants we analyzed were generated by EMS following Jenkins [13] in stocks isoallelic for *dumpy*. Those labeled with a BM in Table 3 are in an isoallelic 2^nd^ chromosome carrying *cn* and *bw* mutant alleles and generated by Brad Marshall, then an undergraduate researcher in the laboratory. Crosses 2A and 2B were performed and mutants were recovered by Michael Guertin are labeled MG in Table 3. Ross MacIntyre produced and recovered *dumpy* mutants from crosses 3 and 4 and are labeled RM in Table 3. In crosses 2, 3, and 4, the 2^nd^ chromosome was marked with either *net* (II, O.O) or *clot* (II, 16.O). These chromosomes were recovered as recombinants from *net dp* ^+^ *clot* /*net^+^ dp^+^ cl^+^* females where the *net* ^+^ *dp* ^+^ *cl* ^+^ chromosome had been isolated from a wild population from Australia, provided by Chip Aquadro, and made isoallelic with crosses to appropriate 2^nd^ chromosome balancer stocks. We confirmed the identity of the *dp^+^* alleles in the *net* and *clot* stocks by a Southern blot which allowed us to analyze the PIGSFEAST repeat number, thus confirming the *dp* allele came from the chromosome from the Australia line (see Carmon et al. [21]). Second, as described below, when amplicons spanning the entire *dumpy* gene from the two strains, *net dp* ^+^ and *dp* ^+^ *clot*, were denatured, reannealed, treated with the Surveyor nuclease, no base pair mismatches were detected, confirming the sequence identity of the wild type *dumpy* alleles.

### Mutagenesis and mutant allele recovery

For the *dumpy* mutant alleles induced with EMS in the *cn bw*, *net* or *clot* chromosomes, we fed males 0.024M EMS for 24 hours following Lewis & Bacher [43] and Jenkins [13]. F1 males were then scored for mosaic or complete *dumpy* mutant phenotypes. Several different crosses were carried out as shown in Table 2.

In crosses 2A and 2B, mutant F1 males were crossed back to *dp* ^ov1^ *cl*; *e* or *net dp* ^ov1^ *cl* females respectively to determine which mutants transmitted a mutant allele, i.e. had a partially or completely mutant gonad. The mating of the phenotypically *dumpy* F1 flies indicated that only 29% transmitted the new mutation. Previous studies [13] found that 35% of the F1 *dumpy* mutants transmitted the mutant allele to their offspring. Mutant F2 males were then mated to In(2LR) CyO, *dp* ^lvI^ *cl*-4 [44]/ In(2LR) Gla females, and the curly winged progeny assessed for their eye color, i.e. either clot or wild type. In cross 2A, when *net cl^+^* males were mutagenized, surviving curly sibs with wild type eyes were mated to establish a stock of the new *dumpy* mutant. In most cases, the newly induced mutant was lethal over the CyO, *dp* ^lvI^ *cl*-*4* chromosome. In these instances, 5–10 single Gla/*dumpy*? males were then backcrossed to CyO, *dp* ^lvI^ *cl-4*/Gla females. Glazed eyed flies from vials with no curly winged clot eyed progeny were then mated to establish the new mutant strain. In cross 2B, when *dp^+^ clot* males were mutagenized, F1 males were crossed to *dp* ^ov1^ *cn bw* females. If the new mutant transmitted, mutant *dumpy* F2 males were crossed to CyO, *dp* ^lvI^ *cl-4*/Gla females and, as in cross 2A, any curly winged clot eyed sibs were mated to recover the new mutant allele in a stock. As was usually the case, if no curly winged clot eyed flies survived, 5–10 single Glazed eyed males were backcrossed to CyO, *dp* ^lvI^ *cl-4*/Gla females and Glazed eyed flies with straight wings from vials with only Glazed or Curly/Glazed progeny were used to set up the mutant stock. In crosses 3 and 4, we hoped to enrich for *dumpy* vortex mutants by incorporating the *e*(*dp* ^v^) mutant [4] on chromosome 3 in the male and female parental stocks. We also crossed F1 males showing complete or mosaic expression of the vortex phenotype to CyO, *dp* ^lvI^ *cl-4*/Gla females. If the curly winged, clot eyed (cross 3) or wild type eyed (cross 4) progeny showed a vortex mutant phenotype, males were backcrossed to CyO, *dp* ^lvI^ *cl-4*/Gla females and either curly winged, dumpy vortex or glazed eyed sibs with straight wings were used to establish a stock of the new “vortex” mutant. Each new *dumpy* mutant we obtained was phenotypically characterized by crossing it to *dp* ^ov1^ flies and to flies carrying Df(2L)ED250, which deletes the entire *dumpy* gene. Thus, for example, a new *dp* ^ol^ mutant would show oblique wings in the F1s from a cross to *dp* ^ov1^, but not mutant vortices, and the flies heterozygous for the new *dumpy* mutation and Df(2L)ED250 would not survive to adulthood.

### Primer design

Primers were designed using Primer3 or a modification of the Primer3 algorithm available at http://flypush.imgen.bcm.tmc.edu/primer/ [45]. An initial set of 85 overlapping primer pairs, each generating an approximately 1kb product, to cover virtually the entire gene, was developed. A second set to overlap these in the region of the gene from PF to PR, as well as additional primers surrounding exon 11 and throughout intron 11, were later developed. Primers were also designed spanning the upstream region of *dumpy* for approximately 25kb and for exon 1. The primer sequences are listed in Table S2.

### Molecular analysis of *dumpy* mutations

We extracted DNA from individuals heterozygous for a mutation and a progenitor second chromosome that was either *cn bw*, *net cl* ^+^ or *net* ^+^ *cl*. For mutants provided by Mary Prout and Jim Fristrom, we used heterozygotes for the mutant and their 2L progenitor chromosome (see Prout et al. [23]) as the source of DNA. PCR on this DNA provided the sequences necessary for the formation of heteroduplexes following denaturation and renaturation of the PCR products. To find the sites of the lesions in the sets of mutants, we pooled several genomic DNAs and amplified aliquots using 1.25U Optimase Polymerase (Transgenomic, Inc.) with 1.25U GoTaq polymerase (Promega) in Optimase Buffer with the primer pairs. Cycling conditions were 94°C for 2 min and 30 cycles of 94°C for 30 sec, 55°C for 30 sec, 72°C for 2 min, followed by 74°C for 5 min. Products were denatured and reannealed, treated with the Surveyor Mutation Detection Kit for WAVE Systems (Transgenomic, Inc.), and injected on the dHPLC WAVE System (Transgenomic, Inc.) using a standard sizing gradient. DNA from the original unmutated isoallelic stocks did not give any mismatched base pairs in any of the initial 85 amplicons but if one of the reannealed DNAs in the mutant pool contains a base pair mismatch, it is cleaved into two fragments which were detected as smaller, discrete peaks following dHPLC. An example of a dHPLC analysis of a cleaved amplicon is shown in Figure 3. Once an amplicon containing a mutation was detected, it was cloned using a TOPO Zero Blunt Cloning Kit (Invitrogen) and at least eight colonies were sequenced to insure the mutant site was identified.

### v2 and ov1

Since the progenitor chromosome for these mutants was not known, DNA from homozygous individuals was extracted. Primer pairs not giving a product were then used for amplification with iProof Polymerase (Bio-Rad) and GC buffer. Cycling conditions were 98°C for 2 min and 35 cycles of 98°C for 5 sec, 63°C for 15 sec, 72°C for 7.5 min, followed by 72°C for 10 min. After visualization on a gel showing the presence of a long insertion, its ends were sequenced to identify the transposon.

### o2 and ovDG2

DNA from homozygous individuals for *dp* ^o2^ and *dp* ^ovDG2^/ Df(2L)ED250 individuals was extracted. Fragments in regions suspected to contain the mutation were amplified by PCR and sequenced.

### lDG82

Balanced lethal flies were crossed to *dp* ^ovDG2^ and DNA was extracted from *dp* ^lDG82^/*dp* ^ovDG2^ F1s since both mutants were made by Dale Grace [15], [17]. Preliminary data indicated the two mutants were induced in the same progenitor chromosome and *dp* ^lDG82^ was subsequently analyzed as above using Surveyor and WAVE analysis.

### RT-PCR

Total RNA was extracted from 3^rd^ instar larvae and S2 cells. RT-PCR was performed using the SuperScript III One-Step RT-PCR System with Platinum *Taq* High Fidelity (Invitrogen). Cycling conditions were 55°C for 30 min, 94°C for 2 min, 40 cycles of 94°C for 15 sec, 55°C for 30 sec, 72°C for 8 min, followed by 72°C for 10 min. Primer pairs spanning putative alternatively spliced exons in various *dumpy* subloci were used as well as a pair spanning the ZP domain. Amplified products were separated in gels and bands excised and sequenced.

### Computer based analyses

Evolutionary comparisons of the 30 nucleotides at the 5′ ends of *dumpy* exons were made in the *dumpy* genes from 7 Drosophila species – *D. melanogaster*, *D. ananassae*, *D. pseudoobscura*, *D. willistoni*, *D. mojavensis*, *D. virilis*, and *D. grimshawi* whose genomes have been sequenced. Analysis of 30 nucleotides was chosen due to the extremely variable length of *dumpy* exons from 54bp to over 13kb. To do this, we aligned the sequences in MEGA 4 and used the nucleotide model to calculate the average pairwise distance with the Kimura 2-parameter correction [46].

## Supporting Information

Table S1 Properties of *dumpy* mutants derived from crosses 1, 2A, 2B, 3, and 4 shown in Table 2 - *indicates complementation with at least one other lethal allele. **oblique score according to Grace [17] in parentheses. The oblique phenotype was tested over *dp* ^ov1^. ***strong = vortices on most of the dorsal thorax, intermed = 2–4 vortices, mild = 1–2 vortices when present.
(0.20 MB DOC)

Table S2 Primers used in this study - *Primers used for RT-PCR products shown in Figures 6 and 7
(0.26 MB DOC)
