# Supplementary material for: A Molecular Analysis of Mutations at the Complex dumpy Locus in Drosophila melanogaster
Source: PLoS One. 2010 Aug 23;5(8):e12319. doi: 10.1371/journal.pone.0012319 (PMC2930355; doi:10.1371/journal.pone.0012319)
Supplement: Table S1 — Properties of dumpy mutants derived from crosses 1, 2A, 2B, 3, and 4 shown in Table 2 - *indicates complementation with at least one other lethal allele. **oblique score according to Grace [17] in parentheses. The oblique phenotype was tested over dp ov1. ***strong = vortices on most of the dorsal thorax, intermed = 2–4 vortices, mild = 1–2 vortices when present. (0.20 MB DOC) [file pone.0012319.s001.doc]

**Table S1** Properties of *dumpy* mutants derived from crosses 1, 2A, 2B, 3, and 4 shown in Table 2.

| *dp* stock | complementing lethal | phenotype | cross | marker | balancer | oblique** | lethal | vortex*** |
| --- | --- | --- | --- | --- | --- | --- | --- | --- |
| 2M1 |  | ol | 1 | cn bw | Gla | o | l | v+ |
| 1D1 |  | ol | 1 | cn bw | Gla | o | l | v+ |
| 2P1 |  | ol | 1 | cn bw | Gla | o | l | v+ |
| 1B5A |  | o | 1 | cn bw | (viable) | o | l+ | v+ |
| 12B1 |  | ol | 1 | cn bw | Gla | o | l | v+ |
| 1C5 |  | olv | 1 | cn bw | Gla | o | l | v |
| 2HA |  | olv | 1 | cn bw | Gla | o | l | v |
| 5B1 |  | olv | l | cn bw | Gla | o | l | v |
| 21C2 |  | olv | 1 | cn bw | Gla | o | l | v |
| 2C1 |  | olv | 1 | cn bw | Gla | o | l | v |
| 21GA |  | olv | 1 | cn bw | Gla | o | l | v |
| 2G1 |  | olv | 1 | cn bw | Gla | o | l | v |
| 1A |  | lv | 2A | net | Gla | o+ | l | v |
| 6a |  | ol | 2A | net | Gla | o(4) | l | v+ |
| 7d |  | olv | 2A | net | Gla | o(5) | l | v(strong) |
| 7e | * | lv | 2A | net | Gla | o+ | l | v(intermed.) |
| 8a |  | olv | 2A | net | Gla | o(4-5) | l | v(intermed.) |
| 9A |  | olv | 2A | net | Gla | o(4-5) | l | v(strong) |
| 9a |  | olv | 2A | net | Gla | o(4-5) | l | v(intermed.) |
| 10A | * | lv | 2A | net | Gla | o+ | l | v(mild) |
| 10B |  | olv | 2A | net | Gla | o(4-5) | l | v(strong) |
| 12b | * | ol | 2A | net | CyO | o(4) | l | v+ |
| 12d |  | ol | 2A | net | Gla | o(4) | l | v+ |
| 13f |  | ol | 2A | net | Gla | o(4-5) | l | v+ |
| 14A |  | ol | 2A | net | Gla | o(3-4) | l | v+ |
| 14a |  | olv | 2A | net | Gla | o(4-5) | l | v(intermed.) |
| 14B |  | o | 2A | net | (viable) | o(1-4) | l+ | v+ |
| 18b |  | ol | 2A | net | Gla | o(4) | l | v+ |
| 23b | * | lv | 2A | net | Gla | o+ | l | v(mild) |
| 27B | * | olv | 2A | net | CyO | o(4) | l | v |
| 29c |  | ol | 2A | net | Gla | o(4) | l | v+ |
| 33b |  | olv | 2A | net | Gla | o(5) | l | v(strong) |
| 34A |  | olv | 2A | net | Gla | o(5) | l | v(strong) |
| 34b |  | olv | 2A | net | Gla | o(4-5) | l | v(strong) |
| 34c |  | ol | 2A | net | Gla | o(4) | l | v+ |
| 36a | * | ol | 2A | net | CyO | o(3) | l | v+ |
| 38a | * | ol | 2A | net | CyO | o(4) | l | v+ |
| 45a |  | olv | 2A | net | Gla | o(5) | l | v(strong) |
| 47A |  | ol | 2A | net | Gla | o(3-5) | l | v+ |
| 47b |  | ol | 2A | net | Gla | o(4-5) | l | v+ |
| 47c |  | olv | 2A | net | Gla | o(4-5) | l | v(strong) |
| 48a |  | olv | 2A | net | Gla | o(3-4) | l | v(strong) |
| 50b |  | ol | 2A | net | Gla | o(4) | l | v+ |
| 52b |  | olv | 2A | net | Gla | o(4-5) | l | v(strong) |
| 56a | * | ol | 2A | net | CyO | o(3-4) | l | v+ |
| 58a |  | ol | 2A | net | Gla | o(4) | l | v+ |
| 58c |  | olv | 2A | net | Gla | o(4-5) | l | v(intermed.) |
| 58c |  | olv | 2A | net | Gla | o(5) | l | v(strong) |
| 60A |  | olv | 2A | net | Gla | o(4-5) | l | v(strong) |
| 61A |  | olv | 2A | net | Gla | o(4-5) | l | v(strong) |
| 61B | ? | olv | 2A | net | Gla | o(4-5) | l | v(strong) |
| 63A |  | olv | 2A | net | Gla | o(4) | l | v(strong) |
| 64b |  | olv | 2A | net | Gla | o(5) | l | v(strong) |
| 65c |  | olv | 2A | net | Gla | o(5) | l | v(intermed.) |
| 65f | * | lv | 2A | net | Gla | o+ | l | v(mild) |
| 67b |  | ol | 2A | net | Gla | o(4) | l | v+ |
| 71a |  | ol | 2A | net | Gla | o(4) | l | v+ |
| 73A | * | lv | 2A | net | Gla | o+ | l | v |
| 2a |  | ol | 2B | clot | Gla | o(3) | l | v+ |
| 6 | * | olv | 2B | clot | CyO | o(4) | l | v |
| 7b |  | ov | 2B | clot | (viable) | o(1-2) | l+ | v+ |
| 11a | * | olv | 2B | clot | CyO | o(4) | l | v |
| 14 |  | lv | 2B | clot | Gla | o+ | l | v(mild) |
| 24B |  | ol | 2B | clot | Gla | o(3) | l | v+ |
| 26a |  | olv | 2B | clot | Gla | o(3-4) | l | v(mild) |
| 27a |  | olv | 2B | clot | Gla | o(4) | l | v(mild) |
| 27b |  | olv | 2B | clot | Gla | o(4) | l | v(intermed.) |
| 29a | * | ol | 2B | clot | CyO | o(3) | l | v+ |
| 29b |  | olv | 2B | clot | Gla | o(4) | l | v(strong) |
| 30b |  | ol | 2B | clot | Gla | o(3-4) | l | v+ |
| 33a |  | olv | 2B | clot | Gla | o(3-4) | l | v(intermed.) |
| 48a | * | olv | 2B | clot | CyO | o(4) | l | v |
| 49A |  | olv | 2B | clot | Gla | o(5) | l | v(intermed.) |
| 55c |  | olv | 2B | clot | Gla | o(3) | l | v(mild) |
| 56a |  | ov | 2B | clot | (viable) | o(2-3) | l+ | v+ |
| 59 | * | olv | 2B | clot | CyO | o(2) | l | v |
| 64c |  | ol | 2B | clot | Gla | o(3) | l | v+ |
| 70b |  | olv | 2B | clot | Gla | o(4-5) | l | v(intermed.) |
| 70c |  | olv | 2B | clot | Gla | o(3-4) | l | v(intermed.) |
| 74a |  | ol | 2B | clot | Gla | o(3) | l | v+ |
| 88a |  | olv | 2B | clot | Gla | o(3) | l | v(mild) |
| 89a |  | olv | 2B | clot | Gla | o(4) | l | v(intermed.) |
| 102A |  | olv | 2B | clot | Gla | o(4-5) | l | v(strong) |
| 104A | * | olv | 2B | clot | Gla | o(3) | l | v(intermed.) |
| 105A | * | olv | 2B | clot | Gla | o(5) | l | v(strong) |
| 108A |  | olv | 2B | clot | Gla | o(4-5) | l | v(strong) |
| 113A |  | olv | 2B | clot | Gla | o(3) | l | v(intermed.) |
| 116A |  | olv | 2B | clot | Gla | o(4-5) | l | v(strong) |
| 116B |  | ol | 2B | clot | Gla | o(3) | l | v+ |
| 120A |  | olv | 2B | clot | Gla | o(5) | l | v(strong) |
| 121A |  | olv | 2B | clot | Gla | o(5) | l | v(strong) |
| 121B |  | ol | 2B | clot | Gla | o(3-4) | l | v+ |
| 124b |  | olv | 2B | clot | Gla | o(4) | l | v(intermed.) |
| 128b |  | olv | 2B | clot | Gla | o(4) | l | v(intermed.) |
| 129b |  | olv | 2B | clot | Gla | o(4) | l | v(intermed.) |
| 131f |  | olv | 2B | clot | Gla | o(4) | l | v(intermed.) |
| 134d |  | olv | 2B | clot | Gla | o(3-4) | l | v(mild) |
| 136A |  | ol | 2B | clot | Gla | o(4) | l | v+ |
| 146 |  | olv | 2B | clot | Gla | o(4) | l | v(strong) |
| A |  | olv | 2B | clot | Gla | o(5) | l | v(strong) |
| B |  | ol | 2B | clot | Gla | o(1) | l | v+ |
| Y | * | olv | 2B | clot | CyO | o(3) | l | v |
| lv-R1 |  | lv | 3 | clot | Gla | o+ | l | v |
| lv-R2 |  | lv | 3 | clot | Gla | o+ | l | v |
| olv-R1 | * | olv | 3 | clot | Gla | o | l | v |
| olv-R3 |  | olv | 3 | clot | Gla | o | l | v |
| olv-R4 |  | olv | 3 | clot | Gla | o | l | v |
| olv-R5 |  | olv | 3 | clot | Gla | o | l | v |
| olv-R6 |  | olv | 3 | clot | Gla | o | l | v |
| olv-R7 |  | olv | 3 | clot | Gla | o | l | v |
| olv-R8 |  | olv | 3 | clot | Gla | o | l | v |
| olv-R9 |  | olv | 3 | clot | Gla | o | l | v |
| olv-R10 |  | olv | 3 | clot | Gla | o | l | v |
| olv-R11 |  | olv | 3 | clot | Gla | o | l | v |
| olv-R12 |  | olv | 3 | clot | Gla | o | l | v |
| 13V | * | olv | 4 | net | Gla | o | l | v |
| A4 | * | olv | 4 | net | Gla | o | l | v |
| A12 |  | ov | 4 | net | (viable) | o | l+ | v |
| B11 | * | olv | 4 | net | Gla | o | l | v |
| B16 | * | olv | 4 | net | Gla | o | l | v |

*indicates complementation with at least one other lethal allele

**oblique score according to Grace [17] in parentheses. The oblique phenotype was tested over *dp*^ov1^

***strong = vortices on most of the dorsal thorax, intermed = 2-4 vortices, mild = 1-2 vortices when present
