# Supplementary material for: A Molecular Analysis of Mutations at the Complex dumpy Locus in Drosophila melanogaster
Source: PLoS One. 2010 Aug 23;5(8):e12319. doi: 10.1371/journal.pone.0012319 (PMC2930355; doi:10.1371/journal.pone.0012319)
Supplement: Table S2 — Primers used in this study - *Primers used for RT-PCR products shown in Figures 6 and 7 (0.26 MB DOC) [file pone.0012319.s002.doc]

**Table S2** Primers used in this study

| Primer Name | Primer Sequence |
| --- | --- |
| 1F | GACCAGGCCAATTGTGTTTT |
| 1R | CTCGGAGTTCCCTCATTCAC |
| 2F | TGGAAGAAAGCCGAGAAAAC |
| 2R | GCTGTGGCTTTTCAATTGCT |
| 3F | GTTGCATGGCGTGAAGTG |
| 3R | GATTTCTCAACCCGATGTGG |
| 4F | CGGCGAACAACTTTTGTTTT |
| 4R | CGCTGTTTGCATTCGTTCT |
| 5F | AACCGTTAAAATCCCCTGCT |
| 5R | GGGTTTTGTGATGCCGTAAT |
| 6F | GACAGTGAGAAACCGGCTGT |
| 6R | CAAAAGACATGCTGGCACAA |
| 7F | GTGTACGCAGCACCAGTAGC |
| 7R | ATGGAGATGGAGATGGAGCA |
| 8F | GTCCTCAAAAGCCCCAAAC |
| 8R | TGCATTTGTGTGCAAAACATT |
| 9F | GAAAGTCATGTCCGCTCGTA |
| 9R | TGACAGCATTCCATGTTTGC |
| 10F | GACTGCCATTTGGAGGAGAC |
| 10R | CACCAGTTGCATACGCTTTG |
| 11F | GAAAATTCCAAACGAAAATGG |
| 11R | CGTCCGTGTTCAAACAATCC |
| 12F | GATACGACGGCGATGGAC |
| 12R | AAGCTGGAAGCTTGCAAAAC |
| 13F | GCCATCCTTGTTTCTTGCTT |
| 13R | TTGTACACGTGGCATTTTGG |
| 14F | GTGTGGACATCGATGAGTGC |
| 14R | TTTGGTAACCCTTGGGACAG |
| 15F | GTTGTGTTCTCTCCGGTTGC |
| 15R | CGCAGATGCACTTTCCATT |
| 16F | CATCAATGGAAGATGCAAGG |
| 16R | TTGATCTTGCACAGCTCGTT |
| 17F | CGTTAACGGCATCAAGGAGT |
| 17R | ACATCCATCCTTGGCAGTGT |
| 18F | CCGGAGGAGTATGCCAAAAT |
| 18R | ACAGAGCTTCGTGTCCTTCC |
| 19F | GTGCCAATAATCTGGCCTGT |
| 19R | GTCTTCGCCACATTCGCTAT |
| 20F | CCCAATTCCAATTCTGTTCG |
| 20R | CCAGTCAAAGGTTGCGTTGT |
| 21F | TGGCACTAAACAGGAGCTTAAC |
| 21R* | ATTGGGCACCATACTCTCCA |
| 22F | TCTGGAAAATCCCTGTGGTC |
| 22R | GCCTCTCTCGCAAAGCTG |
| 23F | GAAAGTGCCGGAATAAGTGC |
| 23R | CAGGGCCGATAGATTAGTGG |
| 24F | CGTTGAAGTTTTTACCCTTACCA |
| 24R | AAGCGTGGCATTATGTGTTG |
| 25F | TAATGACAAATGCCGCGATA |
| 25R | CGTTATTGGCGATTGGATTT |
| 26F | TTGCTATTGCCAGACTTGTACC |
| 26R | AGGGCTCGTTGGGATATTG |
| 27F | CCAATCCCAAATCAACCATT |
| 27R | GATCGACGCACTTGAGCTT |
| 28F | AGAACCACAAGCCAGTCTGC |
| 28R | GGGATATACTTTTGGATGTGTGG |
| 29F | GGCGTTCGTTCCTAATTTGA |
| 29R | GTTCTCTTTTCGCTGGGAGA |
| 42F | ATCTTCTCCGAGCGAAGTGA |
| 42R | CCTGTGGTTCCTTGCAATTC |
| 43F | GACCCGATTGCTCTATGAAAG |
| 43R | TCAAGAATCCTGTTCGGTAGG |
| 44F | CCGTAGCTAAAGCCTGCTAAA |
| 44R | TTCATTTTGGGCGTCGTAGT |
| 45F | ATCACCGATACTCCGACACC |
| 45R | GCTGTTGATGCAAACAGCAT |
| 46F | AGCGTGCATCAACCAACTTT |
| 46R | CGGTGGTTGACCCTCATATT |
| 47F | GTCCCAACTCTGGATGTCGT |
| 47R | GTATGCTGGCGCACATTACA |
| 48F | CGTGCATTAACCACAAGTGC |
| 48R | CTCGAAGTTGGCCTCACAG |
| 49F | GATCCATGCCCAGGTATTTG |
| 49R | GACTTGACATTTGGCGTTGA |
| 50F | TGTGCTCAGCATTTGTCCTG |
| 50R | ATCGCGGCACTTCATGTT |
| 51F | CTGAGTGCGTCCAGAATAGC |
| 51R | GAATATCCGGGCTCACACAT |
| 52F | CAGAATGGCCTGCATCAAT |
| 52R | GAAGGGATCACCGCTGAAG |
| 53F | TCTGCCGCGTAGTCAACC |
| 53R | TCGAGCAGTCACTATTGAGCA |
| 54F | GTTCCTGCTCCTGTCTACCG |
| 54R | GCTTACGTTGTGGGTTACAGC |
| 55F | TTGTAAACCACTCGCCCATC |
| 55R | TAGCCGTGCCAGATAGTTGA |
| 56F | TTAAATCCCCAAAAGCACGA |
| 56R | GTGCACCAATGTAGCCTTCC |
| 57F | GACCGTACAGCCAGTGCTT |
| 57R | AGTTGGGTGGCTGGTTGAT |
| 58F | CTCCATGTGGACCCAACAG |
| 58R | GGGAGCAGGCCTCAATAATA |
| 59F | CTCCTGCGTCAAGGGTTATG |
| 59R | AAGGAGATGGAATGCAAGGA |
| 60F | TGCAACGATGGCTATACTGG |
| 60R | TATGTGGCACAAGCTGTTGG |
| 62F | TCCCCTGTTACCCATGATCT |
| 62R | GGTTCGAAAACCGGACTACTT |
| 63F | ACTTGGCACATTGCACTCAC |
| 63R | TTGATGCGAAAAAGAAGCAA |
| 64F | TCTGTGTCAGTGTCGTCAAGG |
| 64R | GGGATCTCCACATCGTTGAT |
| 65F | ATCAGCAAGCAGTGTGTTCG |
| 65R | GCAGGATGGTGGAGTTCCTA |
| 66F | AGCACCCCAAAACGAGTATG |
| 66R | CCAGACATTTGCTCTGGACA |
| 67F | TTGTGGGGTAAATGCCTTGT |
| 67R | TACAGGTGGCCAAGTGATTG |
| 68F | AATCAGGCATGTGTCAACCA |
| 68R | CAGGCAAGAGCAAACTCCAT |
| 69F | ATCAGTTCGAGACCCACTGC |
| 69R | GGCATTGATTCCACAAGGAC |
| 70F | AAGGCATGGAAGGTGATCC |
| 70R | GTGCAGCTAGGCGTGTGAT |
| 71F | GAATGTCCTAGCAGCCAAGC |
| 71R* | CGAGGGACACTCTGAATTGG |
| 72F | ATGCCTTGGTGATTTTGTCG |
| 72R | TCCCGATAGGGAGCTACATC |
| 73F | CCCCGACCAACAAGTAAGAA |
| 73R | GGACTGTGGTTGACTGTTCG |
| 74F | AAGCTTGGCGTGTCAGAATC |
| 74R | CTGTACATTCCGGTCGACAA |
| 75F | AACCCTCTCCTTGTGGTCCT |
| 75R | GATGGGTAACACGGATCTGG |
| 76F | GTTGCCCAGAGCGTATGAC |
| 76R | ATATGAACGCGGAGCAAACA |
| 77F | TGTCAGAATGGCTTTGTTGG |
| 77R | CACCTCACATATGGCATTGG |
| 78F | GCGTGCGTGAGAAACAAGT |
| 78R | GATATCCCACATGGCAGTTG |
| 79F | GGCTTGTCTCAACCAGCATT |
| 79R | GGCTTTGTCTCTGGCACAGT |
| 80F | AGGACAGTGCTCATGTCTGC |
| 80R | GTAGACACTGGCACGAAGCA |
| 81F | GCGAGAGCAAATCAATCCAT |
| 81R | ATCGGCTACGACGGTAACTG |
| 82F | TCATGTCCCGAGGGTTATTT |
| 82R | GTATGGCAGTTGTGGGAGGT |
| 83F | TCCTGGTTTGGTGGGTAATC |
| 83R | GGTTGCGGAGATGGATAGTT |
| 84F | ATATTCCCTCTGCCCCTCAA |
| 84R | GAAGTGGCACTGATGGAATG |
| 85F | AGGCACCTGTTTACGACGTT |
| 85R | AATCACCCCAGGAGTCAATG |
| 86F | TACAGCCTAAGCCTCCGGTA |
| 86R | AAGCTCTGCACTGGGAATTG |
| 87F | ATCGTACCGCAACGTGCT |
| 87R | TTCGCATTAGGGTCGATTTT |
| 88F | ATGTGGGCAATCCCTATCAC |
| 88R | GCCGCTCCCACATAATTG |
| 89F | GTGGACCTTACTCCCAATGC |
| 89R | GCTTGGCCATTTACATTCGT |
| 90F | CCAGAGTACCAGAACCAGTGC |
| 90R | GAACTCAGGGTGCATTCAGG |
| 91F | GCCGAATTCAGAACGAGAAG |
| 91R | ACTGAAGCCAGCTGGACAA |
| 92F | GAAGACCCGTGCAACTGTG |
| 92R | AGTTCTGTACGCGACACTCG |
| 93F | CACGGCCTGCATCAATAAAC |
| 93R | CACACGGACTGATGCAAATC |
| 94F | CTCGCATCGAATGCTACACT |
| 94R | CATCTTACGGCAAGCTCCAC |
| 95F | TGCTCAGTGCAGTGTCCTAAA |
| 95R | CTTGGAGCACTCAAACTCAGG |
| 96F | ATGCCGAATGCCGTATTAAG |
| 96R | GTGATGCACTGGCACTGG |
| 97F* | AAGGTGTGCGGAGTGAATG |
| 97R* | TGGCATTTGATGAGATTGTGA |
| 98F | CGCTCCATAGCGGATAACTC |
| 98R | GTGTGCGTGTGTCTGTGCTA |
|  |  |
| 42.5F | CCACGAGGACAAGTGACAGA |
| 42.5R | GGAAAATGGCTGAGGATTTG |
| 43.5F | GTTGCCTGCTTTTTGTCTGC |
| 43.5R | ACGGGTGTAACGGGAGGTTA |
| 44.5F | CCAACAACCGGTGCTTGTAT |
| 44.5R | CCGGCGGTTCTACTTGTTAG |
| 45.5F | GGGCTCGTATTCTGGCATTA |
| 45.5R | GTAGGCGTTGCCCAGAAAT |
| 46.5F | ATCAATCCCTGCCAAGAGG |
| 46.5R | CATTGGGTCCACAAGGATTG |
| 47.5F | GGAGATGCTTACCAGGGATG |
| 47.5R | ACCGCAATGGAGTTTAGCAC |
| 48.5F | CGCTTGTCCCACAGGACTAT |
| 48.5R | TGAATTTTGGTAGGGACAGGA |
| 49.5F | CGAGGACTACGAGGGAGATG |
| 49.5R | ACAAATGCCAATGCAAGGAT |
| 50.5F | ACCGAACTGTCGACCTGAAT |
| 50.5R | CTTTGGGCAGGGGTAGGTA |
| 51.5F | GGCCCAACTCGTTATACACC |
| 51.5R | GGATCCACGCACTTTTGATT |
| 52.5F | GGGGATCCATATGTCGAGTG |
| 52.5R | TGTCAAGCAATGATGAAATGG |
| 53.5F | GCCAACTATTCACCGAATCA |
| 53.5R | GGGCTATTTGCACGTTATGA |
| 54.5F | GTTGGGTGGGTCGAATTTTA |
| 54.5R | TCACTGTTCACCACGCATTC |
| 55.5F | ACCCGATGAACGTCTGACTC |
| 55.5R | GGATGCAGGGATTACCAGAC |
| 56.5F | TCAACAACAAGTGCCAGGAT |
| 56.5R | ATGGCATAGTCTCCGCAAAT |
| 57.5F | CGAGAGTGCGAGAATAACGA |
| 57.5R | AAAGCAATCCGCATTTTGTC |
| 58.5F | TTGCACCAAGAAGGAAGAAGA |
| 58.5R | CAATACACTCGGCATTTGGA |
| 59.5F | CTGTGTCCTGCTCTTGTCCA |
| 59.5R | ACGAACTCGAAGCAACGAAC |
| 60.5F | TTCAGAACCACTTGGCCATC |
| 60.5R | CCTTCTGACAGCCCGTAAAC |
| 61.5F* | TGTGTGAATGCCTGTGGATT |
| 61.5R | CAGCAGGGACACGAGGTAAT |
| 62.5F | TTTTTGCCTTTAACCCCTGA |
| 62.5R | ATCGCGACACTTCTGCTGTT |
| 63.5F | ACCCGTATCAGTCGTGTCGT |
| 63.5R | CTGCAGCAACATGTTTGGAT |
| 64.5F | TTTACGGGTGATCCGTTCTC |
| 64.5R | GAGCTGGTCACACACTCTGG |
| 65.5F | CCTGTTCCTGTGTGGAGACC |
| 65.5R | GTCTCAATAATCGGCGCTGT |
| 66.5F | CCACGTAAGCCCATCTCCTA |
| 66.5R | TTGACGACGTTGCATAGAGC |
|  |  |
| 66.5F_08 | TGCCGCAATACTTTGGAGAT |
| 66.5R_08 | TCGAAGGGGGTAAACAATCA |
| 67.5F_08 | GCCTGCATCGATGGTTACAT |
| 67.5R_08 | GGGTCCACACATTTCTGGTT |
| 68.5F_08 | TGCCGACCCGAGTGTATATC |
| 68.5R_08 | CAGGGTGAAGGAACACAAGG |
| 69.5F_08 | GAATTGGTCCTTTCGATACCA |
| 69.5R_08 | AAGCAGCGACAGAATGGACT |
| 70.5F_08 | CCGACCTGAATGCACTACAA |
| 70.5R_08 | ACAGGCGGAGCTCCTACATA |
| 71.5F_08 | AGTGCCGTGAAGTGAATGAA |
| 71.5R_08 | CCATTATATCCCTCCGGACA |
| 72.5F_08 | CCCCAATGCCATTTGTACTG |
| 72.5R_08 | ACAAGGATCACGGCACTTCT |
| 73.5F_08 | TGTCGACCGGAGTGTGTTT |
| 73.5R_08 | TGCAAAAGGGTCTTGGTCTT |
| 74.5F_08 | CCGTGTGATCAGTCACGTTC |
| 74.5R_08 | AAGAAATTGGACAGGCAGGA |
| 75.5F_08 | TGTGGTCCAAACAGCGAAT |
| 75.5R_08 | TTTGAGGTTTTCCACCTTTGA |
|  |  |
| Exon1_F2* | GTCACGTGGATAGTGCTACTACTCTC |
|  |  |
| 5'Dumpy_1F | ATACTCGCTATGCCCGAGTC |
| 5'Dumpy_1R | GCCAACAGGTAGCGAACAAT |
| 5'Dumpy_2F | CACGAGTCTGGAGTCCCAGT |
| 5'Dumpy_2R | AATGCCCTTTATGGCCAAGT |
| 5'Dumpy_3F | TTGGCCAAGTTAGCAGAAGG |
| 5'Dumpy_3R | TAACGAATTTTTCGGCCAAG |
| 5'Dumpy_4F | CTAACGGCACCGTTGACTTC |
| 5'Dumpy_4R | ATGGTGCACTTGCCTCATTA |
| 5'Dumpy_5F | TGACTGCGTGAATCTTGAGG |
| 5'Dumpy_5R | ATCGAAAGGGTTGCCAAAG |
| 5'Dumpy_6F | CGTAGCCAATTGTCACGTTTT |
| 5'Dumpy_6R | CACACCGAGCCAAATATTCTT |
| 5'Dumpy_7F | AACGGCTTTCAACTCGTTTC |
| 5'Dumpy_7R | CCACCAAAACACGACTATTTCA |
| 5'Dumpy_8F | CGGTTTCTAAGCCATCAAGC |
| 5'Dumpy_8R | GCAAATAGAGTCCGCCAATG |
| 5'Dumpy_9F | TTATGCACATGCTCCCAATG |
| 5'Dumpy_9R | CTGTCCCTCCACCGATCTT |
| 5'Dumpy_10F | GCACCTTCAATGCCTCAAAT |
| 5'Dumpy_10R | ATCTCGGCCAGACTCTTTCA |
| 5'Dumpy_11F | AGTGTACAGCGTAGCGACCA |
| 5'Dumpy_11R | TTTTATAAACCTACAGCGGAAGTTG |
| 5'Dumpy_12F | TCAATGGGATAAACCCAACAA |
| 5'Dumpy_12R | TACACCGCAGTTCTGCAAAT |
| 5'Dumpy_13F | AAAGTGGGACGAGAGCAAGA |
| 5'Dumpy_13R | GCCCCACAGATTCTTTCGT |
| 5'Dumpy_14F | GCAGGACAACTCCACTCTGC |
| 5'Dumpy_14R | CTCTTGCCGGTCATAATCGT |
| 5'Dumpy_15F | GCCTGAAACTTGGATCTTGG |
| 5'Dumpy_15R | CACCCAGGTGATGATCAGATT |
| 5'Dumpy_16F | CGCTTCAATATTCTGAGAACCA |
| 5'Dumpy_16R | GCATGCCCATTCTTTCATTC |
| 5'Dumpy_17F | GGTAGCGATATCGGAGCAGA |
| 5'Dumpy_17R | AAAACCCGGCCAGACTTTAG |
| 5'Dumpy_18F | TATTCTGCACGAGACGAAGC |
| 5'Dumpy_18R | CTGGCGAAATCGGTAGATTG |
| 5'Dumpy_19F | GACCAGTCTTTGCCCATTTG |
| 5'Dumpy_19R | TCATTTGTCAGCCAGTTTCG |
| 5'Dumpy_20F | AAGATGAGCCACGAACCAAC |
| 5'Dumpy_20R | AATGGATTTTCGAGCAGTGG |
| 5'Dumpy_21F | GCCATGAAAAAGCACTTCAA |
| 5'Dumpy_21R | TGCAAAGGCGTCTTGGTTAT |
| 5'Dumpy_22F | AACTTTGCTGCCCACACTTT |
| 5'Dumpy_22R | CGGTCCACAGATCTCATCCT |
| 5'Dumpy_23F | AAAGCGAGCCACTAAAAACAA |
| 5'Dumpy_23R | CAAGTTGCTGCTTCTGTTGC |
| 5'Dumpy_24F | ATCTAGGGCCAGCTCTCCAG |
| 5'Dumpy_24R | ATACAGATCTCCCCGCCATT |
|  |  |
| ovF1 | ACCGTTTATGCCACCGAATA |
| ovR2 | AGCAAAAGGTGAGAGCGTGT |
| Intron11_FA1 | GACTGCCAGCCATCCATATC |
| Intron11_RA1 | CGAAAGCTTGATACGGCTACA |
| Intron11_FB1 | TAGTCCTTTGGGCCTTGTCA |
| Intron11_RB1 | CGGAAGCTTCATAAACTATTATACGG |
| Intron11_FC1 | CCTATGATTCTAAATAAACGGTAAAG |
| Intron11_RC1 | ACACTTTACTCCGCCTGCAC |

*Primers used for RT-PCR products shown in Figures 6 and 7
